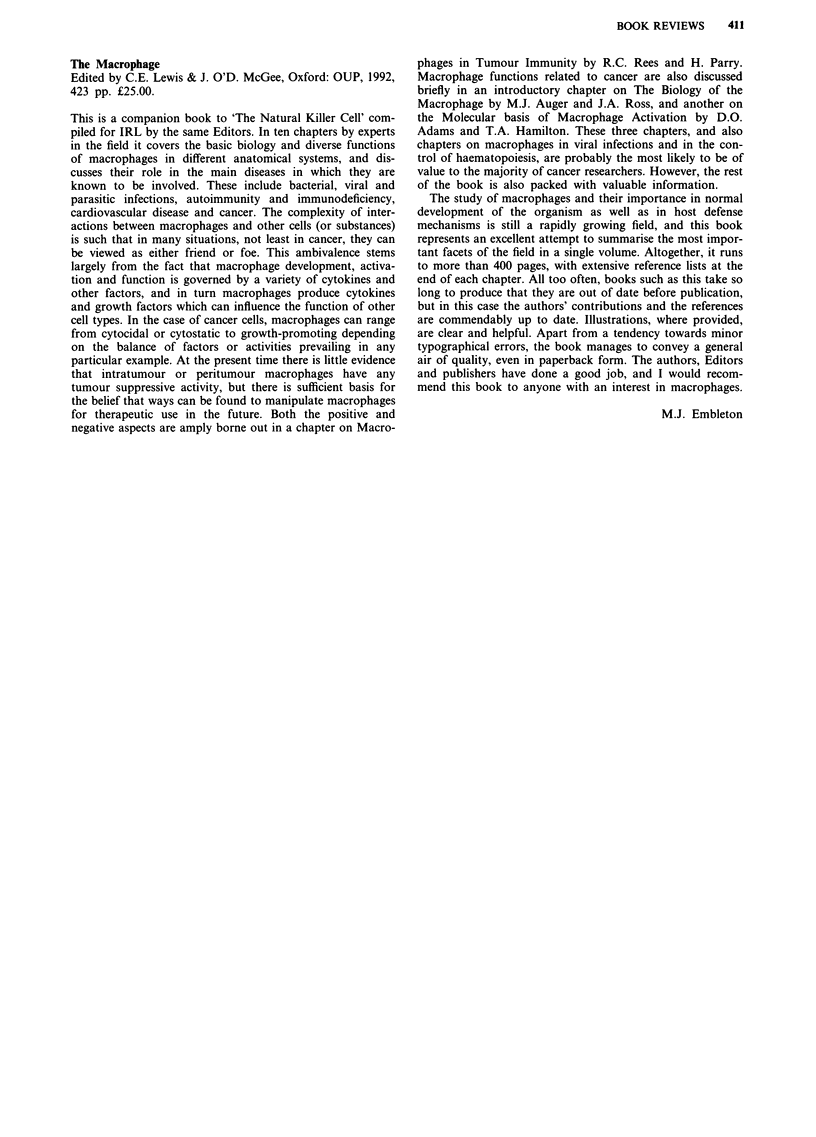# The Macrophage

**Published:** 1993-02

**Authors:** M.J. Embleton


**The Macrophage**
Edited by C.E. Lewis & J. O'D. McGee, Oxford: OUP, 1992, 423 pp. £25.00.

This is a companion book to 'The Natural Killer Cell' compiled for IRL by the same Editors. In ten chapters by experts in the field it covers the basic biology and diverse functions of macrophages in different anatomical systems, and discusses their role in the main diseases in which they are known to be involved. These include bacterial, viral and parasitic infections, autoimmunity and immunodeficiency, cardiovascular disease and cancer. The complexity of interactions between macrophages and other cells (or substances) is such that in many situations, not least in cancer, they can be viewed as either friend or foe. This ambivalence stems largely from the fact that macrophage development, activation and function is governed by a variety of cytokines and other factors, and in turn macrophages produce cytokines and growth factors which can influence the function of other cell types. In the case of cancer cells, macrophages can range from cytocidal or cytostatic to growth-promoting depending on the balance of factors or activities prevailing in any particular example. At the present time there is little evidence that intratumour or peritumour macrophages have any tumour suppressive activity, but there is sufficient basis for the belief that ways can be found to manipulate macrophages for therapeutic use in the future. Both the positive and negative aspects are amply borne out in a chapter on Macrophages in Tumour Immunity by R.C. Rees and H. Parry. Macrophage functions related to cancer are also discussed briefly in an introductory chapter on The Biology of the Macrophage by M.J. Auger and J.A. Ross, and another on the Molecular basis of Macrophage Activation by D.O. Adams and T.A. Hamilton. These three chapters, and also chapters on macrophages in viral infections and in the control of haematopoiesis, are probably the most likely to be of value to the majority of cancer researchers. However, the rest of the book is also packed with valuable information.

The study of macrophages and their importance in normal development of the organism as well as in host defense mechanisms is still a rapidly growing field, and this book represents an excellent attempt to summarise the most important facets of the field in a single volume. Altogether, it runs to more than 400 pages, with extensive reference lists at the end of each chapter. All too often, books such as this take so long to produce that they are out of date before publication, but in this case the authors' contributions and the references are commendably up to date. Illustrations, where provided, are clear and helpful. Apart from a tendency towards minor typographical errors, the book manages to convey a general air of quality, even in paperback form. The authors, Editors and publishers have done a good job, and I would recommend this book to anyone with an interest in macrophages.

M.J. Embleton